# Impact of and Correction for Outcome Misclassification in Cumulative Incidence Estimation

**DOI:** 10.1371/journal.pone.0137454

**Published:** 2015-09-02

**Authors:** Giorgos Bakoyannis, Constantin T. Yiannoutsos

**Affiliations:** Department of Biostatistics, Fairbanks School of Public Health, Indiana University, 410 West 10th Street, Suite 3000, Indianapolis, Indiana 46202, United States of America; FIOCRUZ, BRAZIL

## Abstract

Cohort studies and clinical trials may involve multiple events. When occurrence of one of these events prevents the observance of another, the situation is called “competing risks”. A useful measure in such studies is the cumulative incidence of an event, which is useful in evaluating interventions or assessing disease prognosis. When outcomes in such studies are subject to misclassification, the resulting cumulative incidence estimates may be biased. In this work, we study the mechanism of bias in cumulative incidence estimation due to outcome misclassification. We show that even moderate levels of misclassification can lead to seriously biased estimates in a frequently unpredictable manner. We propose an easy to use estimator for correcting this bias that is uniformly consistent. Extensive simulations suggest that this method leads to unbiased estimates in practical settings. The proposed method is useful, both in settings where misclassification probabilities are known by historical data or can be estimated by other means, and for performing sensitivity analyses when the misclassification probabilities are not precisely known.

## Introduction

Participants in cohort studies are frequently at risk of more than one mutually exclusive event. When observation of one of these events prevents the observation of the others, the situation is said to involve competing risks [1–3]. For example, HIV-infected patients are at risk of dying from AIDS-related and non-AIDS-related causes. Similarly, cancer patients may die from their cancer, from heart disease or other causes [4]. The term “competing risks” also includes non-mutually exclusive events where interest is focused on the first occurring event [2, 3]. When studying changes in combination antiretroviral (cART) regimens in HIV-1 infection, treatment discontinuation and switching to a new regimen are competing risks, even though one event does not preclude observation of the other [5]. In addition, HIV-infected patients may die or disengage from HIV care. While being disengaged from care does not preclude observing a subsequent death, cessation of contact may not allow observation of the death in some cases [6, 7].

A measure estimated from studies with competing events is the cumulative incidence of an event [2, 3], which is particularly useful if interest is focused on the effect of an intervention on a population, on disease prognosis or in cost-effectiveness studies, where risks of multiple events are considered [8]. For example, several studies have explored the extent of patient non-retention in HIV care and treatment programs in a number of settings [7, 9–11]. Patient retention is associated with increased effectiveness of programs, better patient outcomes and reduced risk of subsequent HIV transmission in the community. Estimating the cumulative incidence of disengagement from HIV care can be useful in assessing interventions to increase retention in the presence of all possible patient outcomes. Another example involves assessing the cause of death of oncology patients who may die from complications from their cancer, or from other reasons [4]. Estimating the cumulative incidence of cancer death, in the presence of all other patient outcomes, is a useful measure for assessing patient risk or making decisions about treatment regimens and resource allocation to patient care. Note that this is different from evaluating potential risk factors of an event, in which case, the cause-specific hazard should be used [12].

As complex as analyzing competing-risk data may be, the situation is complicated further when some of the competing events are misclassified. Misclassification happens when one event is mistakenly classified as another among the competing events. For example, deaths from cancer may be misclassified as deaths from other causes and thus, cancer deaths are under-reported [4]. Misclassification can also occur in cases of incomplete death ascertainment because of patient loss from clinic. Biases resulting from incomplete ascertainment of death among HIV-infected patients in both resource-limited [6, 7, 10, 13–15] and replete settings [11] are well understood. What may not be understood as well is that death under-reporting is in fact outcome misclassification as many patients enrolled in clinical programs are considered as disengagers from HIV care because they have died and are no longer observed at their original clinical program [6, 14, 16].

The consequences of misclassification have been extensively studied by several authors. They include biased prevalence and incidence rate estimates [17, 18] and biased relative risk estimates [19, 20]. Additionally, several studies have been carried out to address event misclassification in the context of competing endpoints. Assuming known misclassification rates, Van Rompaye et al. [21] developed an adapted log rank test for the effect of treatment on the cause-specific hazard in clinical trial settings, Van Rompaye et al. [22] proposed a method for the unbiased estimation of the effect parameters in the semi-parametric cause-specific hazards model and Ha and Tsodikov [23] estimated the cause-specific hazard non-parametrically. To our knowledge, the only study to explicitly examine the impact of misclassification on cumulative incidence estimation is the one by Hinchliffe and colleagues involving cancer studies with competing events [4]. That study used simulations to assess the consequences of over and under-recording of cancer as the primary cause of death on death certificates. Thus, to our knowledge, no method has been proposed for unbiased cumulative incidence estimation under misattribution of the cause of failure.

The present study explores the more general mechanism and extent of bias in cumulative incidence estimation due to event misclassification in competing-risk settings. We develop an easy-to-implement and uniformly consistent estimator that can be used to correct for misclassification bias when the misclassification probabilities are known. Our estimator can also be used in settings where the misclassification probabilities are not precisely known, but plausible ranges for these probabilities can also be considered when performing sensitivity analysis. We provide guidelines for the permissible ranges for the misclassification probabilities as a function of the observable outcomes. Such sensitivity analyses would shed light to the direction and potential magnitude of misclassification bias in naïve (i.e. uncorrected for misclassification) cumulative incidence estimates.

To assess the impact of misclassification and evaluate the performance of our methodology, we performed extensive simulations. These show that misclassification can result in seriously biased cumulative incidence estimates, and suggest that the proposed estimator is unbiased when misclassification probabilities are known. We illustrate the proposed method by estimating the cumulative mortality and disengagement from care in a cohort of HIV-infected patients enrolled in a clinical program in sub-Saharan Africa, in a setting where death under-reporting has been extensively documented [6, 14, 24]. We also apply our estimating procedure to sensitivity analyses using cumulative incidence estimates from the study by Hinchiffe and colleagues, which assessed the effect of errors in cause-of-death recording in death certificates on the estimation of cause-specific mortality in cancer patients [4].

## Cumulative Incidence Estimation under Misclassification

We denote the event of interest as *C* = 1 and the competing event(s) as *C* = 2, while *C* = 0 is the situation of being event-free at the end of follow-up. Initially we assume that each event is determined without error. Also, we denote the event time as *T*. In this article, we adopt the classical assumption that right censoring is independent of the event time [1]. The cumulative incidence of the event of interest

$$F_{1}\left(t\right)=\text{Pr}\left(T\leq t,C=1\right)$$

is the cumulative probability of the primary event occurring by time *t*, in the presence of the competing event [1]. It can be estimated non-parametrically by the Aalen-Johansen estimator [25]

$${\hat{F}}_{1}\left(t\right)=\sum_{m:t_{m}\leq t}\frac{d_{1m}}{n_{m}}\hat{S}\left(t_{m-1}\right),$$

where summation is over all observed primary events occurring by *t*, *d*_1m_ and *n*_*m*_ are the numbers of primary events and subjects at risk at time *t*_*m*_ respectively, while *Ŝ*(*t*_*m-1*_) is the Kaplan-Meier estimate of the overall event-free probability before time *t*_*m*_. All subsequently described results hold for any unbiased estimator of the cumulative incidence, not just the Aalen-Johansen estimator. When event classification is subject to error, we observe the possibly misclassified event, *C* ^*obs*^ instead of the true event *C*. If *R* is the indicator of whether event *C* was correctly classified, then, *R* = 1 if *C* ^*obs*^ = *C* and *R* = 0 if *C* ^*obs*^ ≠ *C*. We assume that right-censoring is always correctly ascertained, that is, if patients are observed to be event-free at the end of the study, then they are truly event-free. This is a direct consequence of the assumption of independent right censoring, since mixing cases with an event with censored cases induces association between failure and right censoring time. Based on the observed (but potentially misclassified) event *C* ^*obs*^, one can estimate the *observable* cumulative incidence of the event of interest

$$F_{1}^{obs}\left(t\right)=\text{Pr}\left(T\leq t,C^{obs}=1\right).$$

The true and observed cumulative incidence differ if Pr(*C* ^*obs*^ ≠ *C*) > 0, that is, if there is misclassification.

### Direction of bias resulting from misclassification

It can be shown (see **Observed and true cumulative incidence functions in** S1 Supporting Information) that the observable cumulative incidence of the event of interest is a linear combination of the true cumulative incidence functions for both events:

$$F_{1}^{obs}\left(t\right)=\left[1-a\left(t\right)\right]F_{1}\left(t\right)+b\left(t\right)F_{2}\left(t\right),$$

where *a*(*t*) = Pr(*R* = 0|*C* = 1, *T* ≤ *t*) and *b*(*t*) = Pr(*R* = 0|*C* = 2, *T* ≤ *t*) are probabilities of misclassification of the event of interest and the competing event respectively. For simplicity we assume that the misclassification probabilities are time-constant, i.e., *a*(*t*) = *a* and *b*(*t*) = *b*. The assumption of time-constant misclassification can be relaxed by assuming piecewise constant misclassification probabilities, but to account for this in the context of our estimator (see subsection “Correcting for misclassification bias in cumulative incidence estimation” below) requires some more methodological effort that is beyond the scope of this paper. We note that the misclassification probabilities *a* and *b* are 1 –sensitivity of the diagnostic procedure establishing events 1 and 2 respectively.

If there is no misclassification (i.e. if *a* = 0 and *b* = 0), the observed and true cumulative incidence of the primary event are equal at each time point *t*. Otherwise, two types of misclassification are involved: a) a unidirectional misclassification, and b) a bi-directional misclassification.

In unidirectional misclassification, either only the primary event is misclassified as the competing event (i.e. *C* ^*obs*^ = 2 when in fact *C* = 1) but the secondary event is not misclassified (i.e., *C* = 2 implies *C* ^*obs*^ = 2) so that *a* > 0 but *b* = 0 or vice versa (i.e. *C* = 2 is defined as *C* ^*obs*^ = 1 but *C* = 1 is always recorded as *C* ^*obs*^ = 1) so that *a* = 0 but *b* > 0. It is clear from the definition of the observable cumulative incidence, that if only the event of interest is misclassified, then the observable cumulative incidence $F_{1}^{obs}\left(t\right)$ would be lower that the true cumulative incidence *F*_1_(*t*). Conversely, if only the competing event is misclassified, then the observed cumulative incidence would be higher compared to the true cumulative incidence.

Unidirectional misclassification occurs when estimating the incidence of disengagement from care in HIV patients, particularly in low or middle-income countries [9, 14]. In these settings, many patients who have died (the competing event) are considered as disengagers (the event of interest), that is *b* > 0. However, patients that are truly disengagers cannot be incorrectly classified as having been observed to die, so the event of interest is not subject to misclassification (i.e., *a* = 0). As a result, the observed cumulative incidence estimate of disengagement from care will overestimate the corresponding true cumulative incidence, since

$$F_{1}^{obs}\left(t\right)=F_{1}\left(t\right)+bF_{2}\left(t\right)\geq F_{1}\left(t\right),$$

unless there is no misclassification (i.e., if *b* = 0). The difference between the observed and true cumulative incidence is greater when there is higher under-reporting of mortality and will also depend on *F*_2_(*t*), the true cumulative incidence of death. During periods of high mortality—such as shortly after the initiation of therapy—or among groups at high risk of death, a higher proportion of observed disengagements from care will consist of unreported deaths.

Conversely, unidirectional misclassification of the primary outcome (i.e., *a* > 0 and *b* = 0) leads to underestimation of the corresponding cumulative incidence,

$$F_{1}^{obs}\left(t\right)=\left(1-a\right)F_{1}\left(t\right)<F_{1}\left(t\right).$$

In contrast to the previous situation however, this estimate is dependent only on the magnitude of misclassification and *not* on the cumulative incidence of the competing outcome. In our previous example, the rate of disengagement from care does not enter in the estimation of mortality while on observation.

Bidirectional misclassification arises when both events may be incorrectly classified (i.e. when *a* > 0 and *b* > 0). In these settings, the direction of the bias cannot be a priori determined, since it depends on both misclassification probabilities *a* and *b*. An example of bidirectional misclassification involves the estimation of breast cancer mortality from death certificates [4]. Cause of death information in death certificates is not always accurate [26]. Consequently, some deaths due to breast cancer may be recorded as deaths from other causes, and deaths from other causes may be erroneously classified as breast cancer deaths. The resulting bias is unpredictable. In fact, when both the cumulative incidence and the corresponding misclassification probabilities for the primary and the competing event are equal at each *t* (i.e. when *F*_1_(*t*) = *F*_2_(*t*) and *a* = *b*), the observed and underlying cumulative incidence of the event of interest coincide. While unlikely in practice, this situation underlines the complexity of bias arising from bidirectional misclassification. In most real-world settings involving bidirectional misclassification, the true and observed cumulative incidences are expected to differ. The magnitude and direction of this bias is illustrated in detail by a number of simulation studies later in this article.

## Correcting for Misclassification Bias in Cumulative Incidence Estimation

It can be shown (see **Observed and true cumulative incidence functions in** S1 Supporting Information) that the true cumulative incidence of the primary event can be expressed as a function of the observed cumulative incidence functions:

$$F_{1}\left(t\right)=\frac{1-b}{1-a-b}F_{1}^{obs}\left(t\right)-\frac{b}{1-a-b}F_{2}^{obs}\left(t\right).$$

Consequently, if the misclassification probabilities *a* and *b* are known, the true cumulative incidence of the event of interest can be estimated by:

$${\hat{F}}_{1}\left(t\right)=\frac{1-b}{1-a-b}{\hat{F}}_{1}^{obs}\left(t\right)-\frac{b}{1-a-b}{\hat{F}}_{2}^{obs}\left(t\right),$$ (1)

where ${\hat{F}}_{1}^{obs}\left(t\right)$ and ${\hat{F}}_{2}^{obs}\left(t\right)$ are estimates from the data. These can be estimated by any consistent cumulative incidence estimator, as the Aalen-Johansen estimator. The estimator (1) is not strictly monotonic. That is, the cumulative incidence estimate may decrease slightly in a narrow time interval. To overcome this issue we adopt isotonic estimation [23], that is

$${\hat{F}}_{1}^{m}\left(t\right)=\underset{0\leq \tau \leq t}{\text{max}}{\hat{F}}_{1}\left(\tau \right).$$ (2)

The corrected monotonic cumulative incidence estimator at time *t* is equal to the maximum cumulative incidence for all points with an observed event from any time up to *t*. A key assumption of the estimator is that the sum of the two misclassification probabilities must be less than 1, or equivalently, that the sum of the sensitivities for the two outcomes is higher than 1. In **Uniform consistency of the estimator in** S1 Supporting Information we show that the corrected and monotonic cumulative incidence estimator is uniformly consistent for the true cumulative incidence for the corresponding cause of failure, that is

$$\underset{t\in \left[0,\text{max}\left(t_{i}\right)\right]}{\text{sup}}\left|{\hat{F}}_{1}^{m}\left(t\right)-F_{1}\left(t\right)\right|\overset{p}{\to }0,$$

where max(*t*_*i*_) is the maximum follow-up time. Bootstrap methodology can be applied for the calculation of pointwise confidence intervals [27]. Implementing the proposed estimator only requires standard software that implements the Aalen-Johansen estimator.

## Sensitivity Analyses

In studies where misclassification is suspected, but the misclassification probabilities are unknown, we can use estimator (1) with a range of plausible values for *a* and *b* to perform sensitivity analyses. It turns out that the ranges of possible values for misclassification probabilities are bounded by

$$0\leq a<1-\frac{F_{1}^{obs}\left(t\right)}{1-S\left(t\right)}$$

for the misclassification probability of the primary event and

$$0\leq b<\frac{F_{1}^{obs}\left(t\right)}{1-S\left(t\right)}$$

for the misclassification probability of the competing event, where $F_{1}^{obs}\left(t\right)$ is the observed cumulative incidence of the primary event and *S*(*t*) is the overall survival probability (see **Mathematically permissible ranges for misclassification probabilities in** S1 Supporting Information). Thus, only part of the entire zero-one range need be considered (see supporting information and the illustration of the method below).

## Simulation Studies

To evaluate the direction and magnitude of the misclassification bias when using the Aalen-Johansen estimator, and to evaluate the performance of the proposed estimator, we carried out several simulation studies involving both unidirectional and bidirectional misclassification. Misclassification probabilities ranged from 0% (no misclassification) to high (30%), and hazard rates were assumed to be moderate (0.5) for the primary endpoint and for the competing event were assumed low (0.25), moderate (0.5) or high (1) depending on the scenario. In total, 45 simulation scenarios were considered for all possible combinations of misclassification probabilities and hazard rates for the two events. For each scenario 1,000 datasets were generated, each consisting of 1,000 subjects. More details on the simulation study design are given in the **Simulation study design in** S1 Supporting Information.

### Simulation studies under unidirectional misclassification

**Fig 1:**
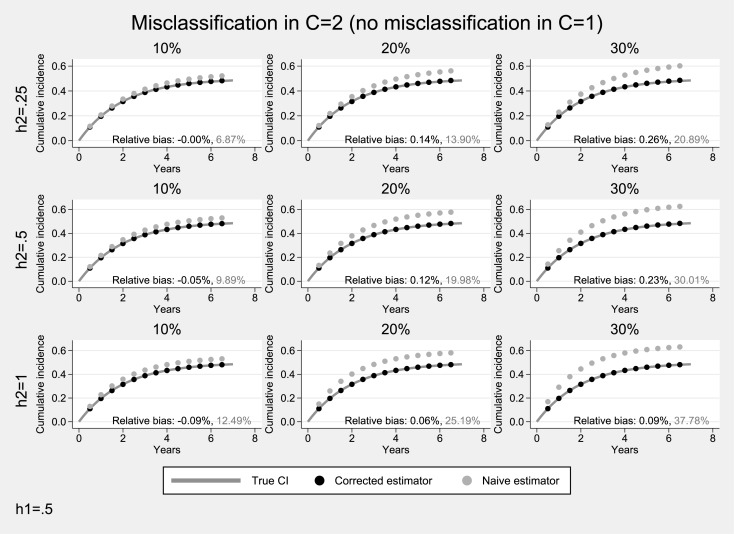
Simulation results for the case of unidirectional misclassification: Only the competing endpoint was subjected to misclassification. The hazard for the primary endpoint was assumed to be moderate (*h*_1_ = 0.5). Solid lines correspond to the true cumulative incidence whereas dotted lines to the estimates from the non-parametric Aalen-Johansen estimator for the primary endpoint.

**Fig 2:**
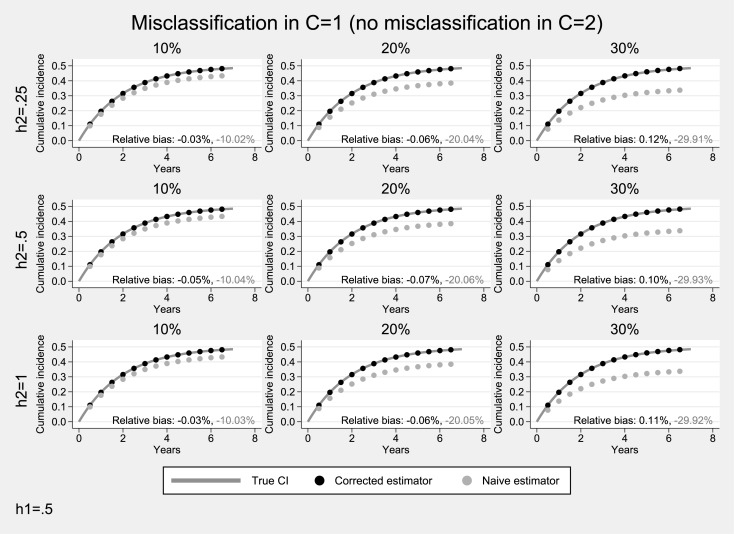
Simulation results for the case of unidirectional misclassification: Only the primary endpoint was subjected to misclassification. The hazard for the primary endpoint was assumed to be moderate (*h*_1_ = 0.5). Solid lines correspond to the true cumulative incidence whereas dotted lines to the estimates from the non-parametric Aalen-Johansen estimator for the primary endpoint.

Results from simulations under unidirectional misclassification and a moderate hazard (0.5) for the event of interest, are presented in Figs 1 and 2. When only the competing event is misclassified (Fig 1), the estimated cumulative incidence systematically overestimates the underlying cumulative incidence of the primary event (average relative bias: 7%-38%). As expected, the degree of overestimation was higher when misclassification was more pronounced and the hazard of the competing event was higher. In contrast, the proposed estimator performed very well (average relative bias <1%). When only the primary event was subject to misclassification (Fig 2), there was a systematic underestimation of the cumulative incidence of the primary event (average relative negative bias: 10%-30%). Bias was more pronounced when the misclassification probability of the primary event was higher but did not depend on the incidence of the competing event. The proposed estimator resulted in practically unbiased estimates (average relative bias: <1%).

### Simulation studies under bidirectional misclassification

**Fig 3:**
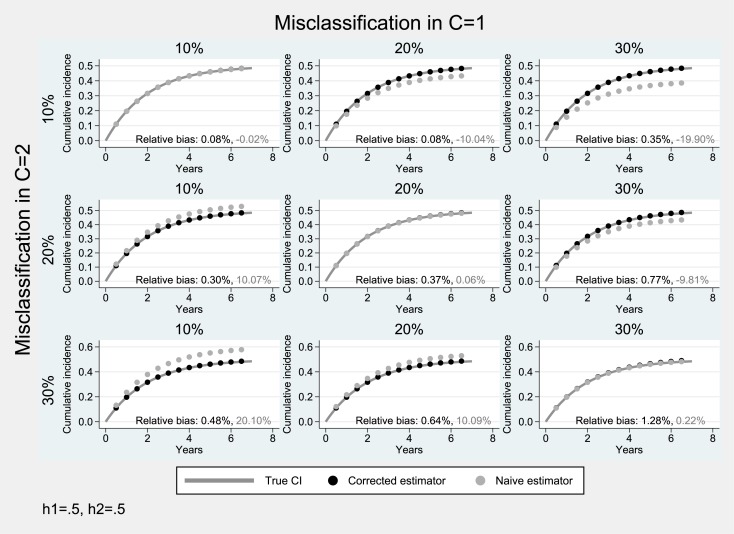
Simulation results for the case of bidirectional misclassification: Hazard parameter for the primary endpoint equal to that of the competing event (*h*_1_ = *h*_2_ = 0.5). Solid lines correspond to the true cumulative incidence whereas dotted lines to the estimates from the non-parametric Aalen-Johansen estimator for the primary endpoint.

**Fig 4:**
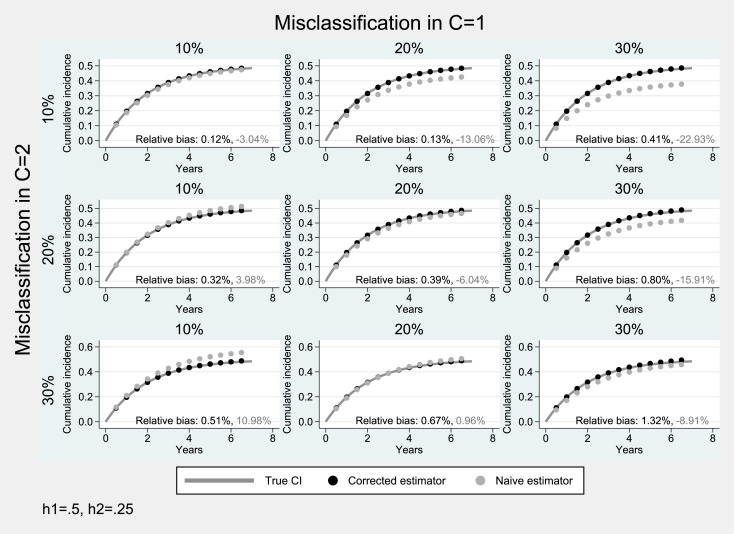
Simulation results for the case of bidirectional misclassification: Hazard parameter for the primary endpoint greater than that of the competing event (*h*_1_ = 0.5, *h*_2_ = 0.25). Solid lines correspond to the true cumulative incidence whereas dotted lines to the estimates from the non-parametric Aalen-Johansen estimator for the primary endpoint.

**Fig 5:**
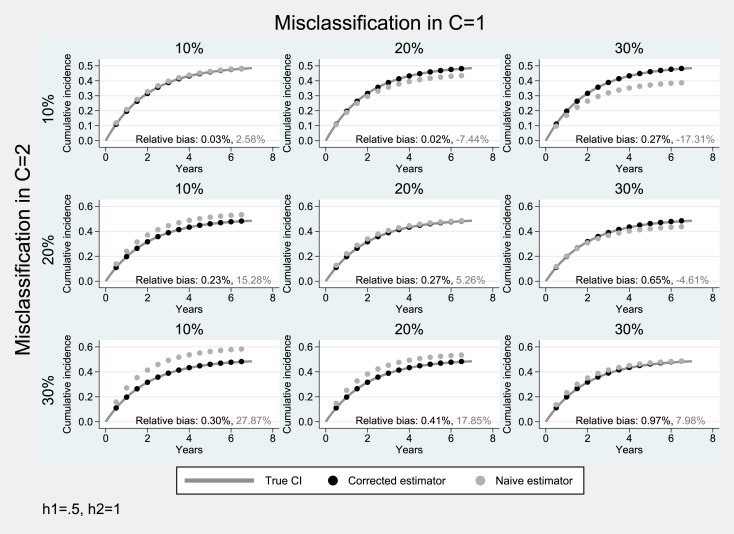
Simulation results for the case of bidirectional misclassification: Hazard parameter for the primary endpoint lower than that of the competing event (*h*_1_ = 0.5, *h*_2_ = 1). Solid lines correspond to the true cumulative incidence whereas dotted lines to the estimates from the non-parametric Aalen-Johansen estimator for the primary endpoint.

Simulation results from scenarios with bidirectional misclassification and moderate hazard of the primary event (0.5) are presented in Figs 3–5. When the cumulative incidence of the two events is equal (Fig 3), the misclassification bias tends to cancel out (diagonal panels of Fig 3) as expected. When the misclassification probability for the primary event is higher than that of the competing event (*a* > *b*), there is an underestimation of the cumulative incidence of interest (average relative negative bias: 10%-20%), while in the converse case, (*a* < *b*), the estimated cumulative incidence of the event of interest is upwardly biased (average relative bias: 10%-20%). In both scenarios, the absolute degree of bias depends on the difference between the two misclassification probabilities *a*–*b*. In all scenarios of Fig 3, the proposed estimator produced approximately unbiased estimates (average relative bias: ≤1%).

In scenarios where the cumulative incidence of the competing event is lower than that of the event of interest (*F*_1_(*t*) > *F*_2_(*t*)), there is an additional tendency for underestimation (diagonal panels of Fig 4 where the two events were subject to the same degree of misclassification *a* = *b*). In these cases the performance of the naïve cumulative incidence estimator was poor (average relative bias: 3%-23%), as opposed to that of the proposed estimator (average relative bias: ≤1%).

When the cumulative incidence of the competing event is higher than that of the event of interest (i.e., *F*_1_(*t*) < *F*_2_(*t*)), there is an additional tendency for overestimation (diagonal panels of Fig 5 where the two events were subject to the same degree of misclassification *a* = *b*). The naïve cumulative incidence estimator produced biased estimates (average relative bias: 3%-28%), whereas the proposed estimator was practically unbiased (average relative bias: ≤1%).

## Illustration

We illustrate the use of our estimator using data from a program evaluation in HIV care and treatment in resource-limited settings [14]. We also provide an example using the estimator in a sensitivity analysis following the assumptions in the study described by Hinchliffe and colleagues [4].

### Correcting for misclassification when misclassification probabilities are known

We use patient data from an HIV care and treatment program in sub-Saharan Africa as described by Yiannoutsos et al. [14]. We aim to estimate cumulative mortality at one year from cART initiation and the risk of patient disengagement from HIV care while correcting for the misclassification due to death under-recording. The naïve estimate of the cumulative incidence of death at one year after enrollment was 2.95% and 0.24% for patients with CD4<100 cells/μL and CD4≥350 cells/μL at enrollment respectively. The corresponding figures for disengagement from care were 42.89% and 33.24%. Based on a sample of 621 lost patients whose vital status was subsequently ascertained through active outreach, it was found that within 30 days from the last clinic visit, 42 patients were actually dead. The 30-day criterion was used to define early deaths in the sense proposed by Geng et al. [7]. Consequently, the estimated probability of an early death among those that were initially considered as disengagers from HIV care is 42/621 = 0.07. The estimated probability of misclassification, that is of observing a disengagement from care among dead patients can be estimated using the Bayes theorem as:

$$\frac{\frac{3522}{8977}0.07}{\frac{3522}{8977}0.07+\frac{106}{8977}1.0}=0.7,$$

Where 3,522 are the number of the observed disengagements from care, 106 are the observed deaths, and 8,977 the total sample size. Note that the misclassification is this setting is unidirectional since the probability of disengagement from care given an observed death is 0. Based on this estimate of the misclassification probability and assuming that it is constant over time we can apply the proposed estimator to adjust for misclassification. As mentioned above, the probability of incorrectly recording as dead a patient who is disengaged from care is 0. Consequently, the corrected estimates for mortality at 1 year from enrollment are $\frac{0.0295}{1-0.7}=0.0983$ (9.83%) and $\frac{0.0024}{1-0.7}=0.0080$ (0.80%) for patients with CD4 count<100 cells/μL and >350 cells/μL respectively. The corresponding estimates for disengagement from care are $0.4289-\frac{0.7}{1-0.7}0.0983=0.20$ (20%) and $0.3324-\frac{0.7}{1-0.7}0.0080=0.31$ (31%). Note that the sum of the cumulative incidence of both events is constant between the naïve and corrected quantities.

### Sensitivity analysis

**Fig 6:**
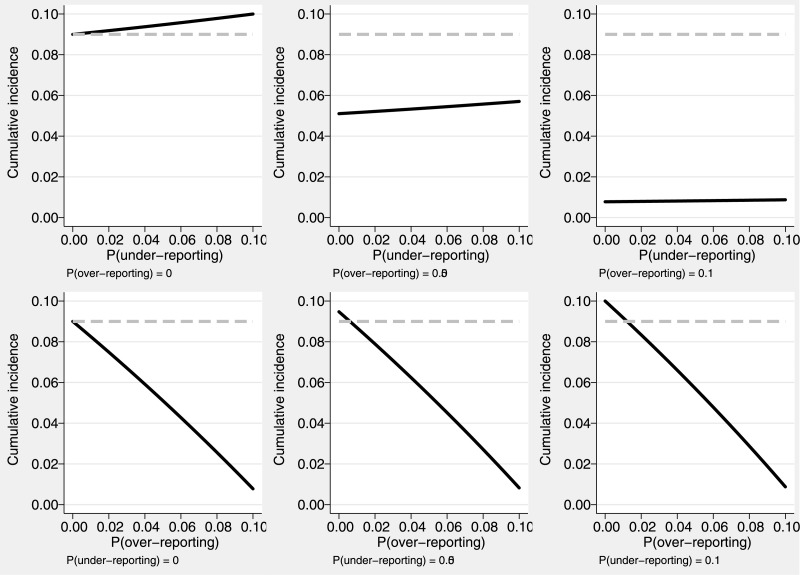
Sensitivity analysis regarding the effect of the potential misclassification of cancer. The cumulative incidence of the observed cancer deaths at 10 years after cancer diagnosis was 0.09 (grey dashed lines). The corrected cumulative incidence estimates (solid black lines) are presented according to the different potential levels of cancer death under-reporting (first row) and over-reporting (second row).

The use of our estimator in sensitivity analyses is illustrated using the example of cancer death estimation using death certificates [4]. Although the estimates of cancer mortality and mortality from other causes were not based on the Aalen-Johansen estimator in that study, we can still use the formula (1) for true cumulative incidence in the previous section to perform sensitivity analyses under a range of misclassification probabilities. The estimated, potentially biased due to misclassification, cumulative incidence of cancer death at 10 years after cancer diagnosis for patients aged over 85 years was 9%. The corresponding figure for death from cardiovascular or other causes in this age group was 74%. Here, *a* is the probability of a cancer death reported as death from other causes, and *b* the probability of reporting non-cancer deaths as deaths from cancer on the death certificates. The probability *b* of over-reporting cancer deaths, can be only as high as 10.84% since the observable cumulative incidence is relatively low (refer to the previous for limits on permissible probabilities of misclassification). Results from the sensitivity analysis are presented in Fig 6. Under 5% over-recording and 5% under-recording of cancer death (i.e., if *a* = 0.05 and *b* = 0.05) the underlying cancer cumulative incidence is 5.4%. If over-reporting of cancer deaths is 10% (i.e., if *a* = 0.05 and *b* = 0.10), the true cancer mortality could be as low as 0.8%. If the probability of under-reporting were higher (i.e., if *a* = 0.10 and *b* = 0.05), then an estimate of the true cumulative incidence of death from cancer would be 5.7%. It is thus clear that the estimate of the cumulative mortality from cancer is sensitive to the degree of over-reporting and not on the magnitude of under-reporting of cancer death and also that the uncorrected cumulative incidence of cancer death is, under the values for the misclassification probabilities we assumed, overestimated as a result of misclassification in death certificates.

## Discussion

This study assesses the mechanism of misclassification bias in cumulative incidence estimation under competing risks and proposes a uniformly consistent estimator for the cumulative incidence function when misclassification probabilities are a-priory known. In general, the bias of the naive cumulative incidence estimator for an event in the presence of competing risks depends on both the degree of misclassification and the incidence of the competing event. In unidirectional misclassification, the bias can be either positive or negative depending on which event was misclassified (positive when the competing event is misclassified and negative when the primary event is misclassified). However, when bidirectional misclassification is present, neither the magnitude nor the direction of the bias can be a-priori determined, since they depend on both the misclassification probabilities and the difference in the cumulative incidence of the primary and the competing event.

Through a number of simulation studies, our proposed estimator was shown to be practically unbiased in plausible misclassification scenarios, where the sum of the misclassification probabilities is less than 1, or equivalently, when the sum of the sensitivities associated with each event is higher than 1. This is virtually guaranteed in well-designed studies or studies using diagnostic procedures with sensitivity exceeding 50% for each endpoint so that their sum is greater than 1.

To our knowledge, the only study to directly examine misclassification in this context is the one by Hinchliffe et al. [4]. That study however explored a narrower problem involving over-estimation and under-estimation of cancer-related death in death certificates. By contrast, our study explores misclassification in more general settings and provides mathematical insight on the structure of the bias in unidirectional and bidirectional misclassification. Also, while previous research has addressed various aspects of outcome misclassification in competing risk settings [21–23], the issue of unbiased estimation of the cumulative incidence has not been previously investigated. One potential extension of our work could be the incorporation of time-dependent misclassification probabilities. One potential estimator of the cumulative incidence under time-dependent misclassification would be to use Ha and Tsodikov’s [23] corrected cumulative cause-specific hazard estimator ${\hat{\Lambda }}_{1}^{S}\left(t\right)$ and the Kaplan-Meier estimator for the overall survival Ŝ(*t*) to obtain the corrected cumulative incidence estimator as:

$${\hat{F}}_{1}\left(t\right)=\int_{t}^{0}\hat{S}\left(u-\right)d{\hat{\Lambda }}_{1}^{S}\left(u\right).$$

However, in practice it is not possible to have a-priory knowledge of the misclassification probability at each time point, so we suspect that such an estimator may have limited practical utility and, for this reason, we have not considered it further in this paper.

Our estimator can also be used when misclassification probabilities are not known in advance such as, for example, in sensitivity analyses, when ranges of plausible misclassification rates can be considered. In the problem of death under-reporting in HIV care and treatment programs in low or middle-income settings, a number of papers [14, 15, 24, 28] can provide plausible estimates of death under-reporting in similar settings. In the study by Hinchliffe and colleagues [4] on the other hand, consideration of various levels of misclassification showed that cancer mortality estimation is more sensitive to over-recording of cancer-related causes of death. From this example it was clear that even a small level of misclassification in a competing event with high cumulative incidence can induce high levels of bias.

In all cases, caution should be exercised when using externally derived estimates of misclassification as misclassification can be highly contextual. As guidance, we have derived bounds of ranges for plausible misclassification probabilities (see **Mathematically permissible ranges for the misclassification probabilities in** S1 Supporting Information) based on the fact that the sum of the true cumulative incidences cannot exceed the sum of the observed cumulative incidences.

Our observations on misclassification bias highlight the importance of accurate classification in studies with competing risks. If this is not practically possible, our method can provide corrected cumulative incidence estimates, elucidate the consequences of misclassification and incorporate expert knowledge to improve the resulting estimates, both when misclassification probabilities are a-priory known and when they are unknown, in which case undertaking sensitivity analyses would shed light in the potential magnitude and direction of bias in the naïve estimator.

## Supporting Information

S1 Supporting Information Mathematical derivations and simulation study design. Relation between the observable and true cumulative incidence functions. Proof of the uniform consistency of the corrected estimator. Mathematically permissible ranges for the misclassification probabilities. Simulation study design.
(DOCX)

S1 Data Illustration dataset. Dataset used for the illustration of the corrected cumulative incidence estimator.
(DTA)
