# Supplementary material for: Impact of and Correction for Outcome Misclassification in Cumulative Incidence Estimation
Source: PLoS One. 2015 Sep 2;10(9):e0137454. doi: 10.1371/journal.pone.0137454 (PMC4558089; doi:10.1371/journal.pone.0137454)
Supplement: S1 Supporting Information — Relation between the observable and true cumulative incidence functions. Proof of the uniform consistency of the corrected estimator. Mathematically permissible ranges for the misclassification probabilities. Simulation study design. (DOCX) [file pone.0137454.s001.docx]

**SUPPORTING INFORMATION**

**Proofs**

*Observed and true cumulative incidence functions*

Initially we are going to prove the relation between the observed cumulative incidence of the event of interest and the true cumulative incidence of the two endpoints. Based on the total probability theorem, the observable cumulative incidence of the primary endpoint, under the potentially misclassified event $C^{obs}$, can be expressed as:

$$F_{1}^{obs}\left( t \right)=\text{Pr}\left( T\leq t,C^{obs}=1 \right)$$

$=\sum_{j=1}^{2} \sum_{r=0}^{1} \text{Pr}\left( C=j,R=r \right)\text{Pr}\left( T\leq t,C^{obs}=1|C=j,R=r \right)$

$=\text{Pr}\left( C=1,R=1 \right)\text{Pr}\left( T\leq t,C^{obs}=1|C=1,R=1 \right)+$

$\text{ }\text{Pr}\left( C=2,R=0 \right)\text{Pr}\left( T\leq t,C^{obs}=1|C=2,R=0 \right)$, (A.1)

since if {*C =* 2, *R* = 1} or {*C =* 1, *R* = 0} then Pr(*T* ≤ *t*, *C^obs^* *=* 1|*C* = *j*, *R* = *r*) = 0.

If $\{C=1, R=1\}$ or $\{C=2, R=0\}$ (i.e., if the correct event of interest or the misclassified ancillary event is observed) then it follows that:

$$\text{Pr}\left( T\leq t,C^{obs}=1|C=j,R=r \right)=\text{Pr}\left( T\leq t|C=j,R=r \right)\text{Pr}\left( C^{obs}=1|T\leq t,C=j,R=r \right)$$

$$=\text{Pr}\left( T\leq t|C=j,R=r \right)$$

$$=\frac{\text{Pr}\left( T\leq t,C=j,R=r \right)}{\text{Pr}\left( C=j,R=r \right)}$$

$$=\frac{\text{Pr}\left( T\leq t,C=j \right)\text{Pr}\left( R=r|T\leq t,C=j \right)}{\text{Pr}\left( C=j,R=r \right)}$$

$$=\frac{F_{j}\left( t \right)\text{Pr}\left( R=r|C=j \right)}{\text{Pr}\left( C=j,R=r \right)}, (A.2)$$

where $F_{j}(t)$ is the underlying true cumulative incidence of the endpoint $C=j$ ($j=1,2)$. Note that in the above relation we considered that $\text{Pr}\left( C^{obs}=1|T\leq t,C=1,R=1 \right)$ = 1 and $\text{Pr}\left( C^{obs}=1|T\leq t,C=2,R=0 \right)$ = 1. This is true by definition, since if (a) the true event is the event of interest and there is no misclassification or (b) the true event is the competing event and under misclassification, then it is certain that $C^{obs}=1$ . Substituting Equation (A.2) in Equation (A.1) we have:

$$F_{1}^{obs}\left( t \right)=\text{Pr}\left( C=1,R=1 \right)\frac{F_{1}\left( t \right)\text{Pr}\left( R=1|C=1 \right)}{\text{Pr}\left( C=1,R=1 \right)}\text{Pr}\left( C=2,R=0 \right)\frac{F_{2}\left( t \right)\text{Pr}\left( R=0|C=2 \right)}{\text{Pr}\left( C=2,R=0 \right)}$$

$$=F_{1}\left( t \right)\text{Pr}\left( R=1|C=1 \right)+F_{2}\left( t \right)\text{Pr}\left( R=0|C=2 \right)$$

$=\left( 1-a \right)F_{1}\left( t \right)+bF_{2}\left( t \right),$ $(A.3)$

where $a=\Pr\left( R=0 | C=1 \right)$ and $b=\Pr\left( R=0 | C=2 \right)$ are the probabilities of misclassification under the event of interest and the competing event, respectively.

Further, we need to provide justification for our estimator. For this, we define the true cumulative incidence of the cause of interest in terms of the observable cumulative incidences of the two endpoints. Using the same arguments as in the previous proof we conclude that:

$$F_{2}^{obs}\left( t \right)=\left( 1-b \right)F_{2}\left( t \right)+aF_{1}\left( t \right)\Leftrightarrow$$

$F_{2}\left( t \right)=\frac{F_{2}^{obs}\left( t \right)-aF_{1}\left( t \right)}{1-b}.$ $(A.4)$

Replacing (A.4) in (A.3) we have:

$$F_{1}^{obs}\left( t \right)=\left( 1-a \right)F_{1}\left( t \right)+\frac{b}{1-b}F_{2}^{obs}\left( t \right)-\frac{b}{1-b}aF_{1}\left( t \right)$$

$$=\left[ 1-a-\frac{b}{1-b}a \right]F_{1}\left( t \right)+\frac{b}{1-b}F_{2}^{obs}\left( t \right).$$

Solving the above equation with respect to $F_{1}\left( t \right)$ leads in

$$F_{1}\left( t \right)=\frac{1-b}{1-a-b}F_{1}^{obs}\left( t \right)-\frac{b}{1-a-b}F_{2}^{obs}\left( t \right).$$

Since *a* and *b* are a-priory known we can estimate the observable cumulative incidences by standard methods like the Aalen-Johansen estimator to obtain a corrected estimator for the cumulative incidence of the event of interest (i.e. C=1):

$$\hat{F}_{1}\left( t \right)=\frac{1-b}{1-a-b}\hat{F}_{1}^{obs}\left( t \right)-\frac{b}{1-a-b}\hat{F}_{2}^{obs}\left( t \right).$$

*Uniform consistency of the estimator in (1)*

Next, we derive the uniform consistency of the proposed monotonic estimator of the cumulative incidence $\hat{F}_{1}^{m}\left( t \right)\equiv\sup_{x\leq t} \left[ \hat{F}_{1}\left( x \right) \right]$ where $\hat{F}_{1}\left( x \right)$ is the non-monotonic corrected estimator. Let $\left\| f\left( t \right) \right\|_{\infty}=\sup_{t\in[0,\max\left( t \right)]} \left| f\left( t \right) \right|$ denote the supremum norm of the function of time $f\left( t \right)$, where $\max\left( t \right)$ is the finite maximum follow-up time. Also, denote $w_{1}\equiv\frac{1-b}{1-a-b}$ and $w_{2}\equiv\frac{b}{1-a-b}$ where, as before, $a$ and $b$ are the misclassification probabilities for the main and competing event respectively. We first prove the uniform consistency of the initial, non-monotonic in finite samples, estimator under the assumption of known misclassification probabilities and thus known $w_{1}$ and $w_{2}$. Consider $A_{n}\equiv\left\| \hat{F}_{1}\left( t \right)-F_{1}\left( t \right) \right\|_{\infty}.$ This is independent of $t$ since it represents the supremum over all times $t$. Then, from above,

$$A_{n}=\left\| w_{1}\hat{F}_{1}^{obs}\left( t \right)-w_{2}\hat{F}_{2}^{obs}\left( t \right)-w_{1}F_{1}^{obs}\left( t \right)+w_{2}F_{2}^{obs}\left( t \right) \right\|_{\infty}=\left\| w_{1}\left[ \hat{F}_{1}^{obs}\left( t \right)-F_{1}^{obs}\left( t \right) \right]+w_{2}\left[ F_{2}^{obs}\left( t \right)-\hat{F}_{2}^{obs}\left( t \right) \right] \right\|_{\infty}\leq{\left| w_{1} \right|\left\| \hat{F}_{1}^{obs}\left( t \right)-F_{1}^{obs}\left( t \right) \right\|}_{\infty}+{\left| w_{2} \right|\left\| F_{2}^{obs}\left( t \right)-\hat{F}_{2}^{obs}\left( t \right) \right\|}_{\infty} ={w_{1}\left\| \hat{F}_{1}^{obs}\left( t \right)-F_{1}^{obs}\left( t \right) \right\|}_{\infty}+{w_{2}\left\| \hat{F}_{2}^{obs}\left( t \right)-F_{2}^{obs}\left( t \right) \right\|}_{\infty}\equiv B_{n} , (A.5)$$

where $\left| w_{j} \right|=w_{j}$, $j=1,2$, by the fact that the weights are non-negative real numbers. By the uniform consistency of the usual Aalen-Johansen estimator (Andersen et al., 1996) (under standard regularity conditions) we have that

$$\left\| \hat{F}_{j}^{obs}\left( t \right)-F_{j}^{obs}\left( t \right) \right\|_{\infty}\underset{\to}{p}0, j=1,2.$$

Using Slutsky’s theorem, and the fact that $w_{1}$ and $w_{2}$ are by definition finite real numbers, it follows that $B_{n}\underset{\to}{p}0.$ By inequality (A.5) and the monotonicity of the probability measure it follows that

$$P\left[ A_{n}>\varepsilon\right]\leq P\left[ B_{n}>\varepsilon\right],$$

for every $\varepsilon>0$ and $n$ large enough, and thus, by the consistency of $B_{n}$,

$$\limsup_{n\to\infty} P\left[ A_{n}>\varepsilon\right]\leq\limsup_{n\to\infty} P\left[ B_{n}>\varepsilon\right]=\lim_{n\to\infty} P\left[ B_{n}>\varepsilon\right]=0$$

$\forall\varepsilon>0$ and thus

$$\left\| \hat{F}_{1}\left( t \right)-F_{1}\left( t \right) \right\|_{\infty}\underset{\to}{p}0$$

proving the uniform consistency of the non-monotonic estimator. To complete the proof, we need to show the uniform consistency of the monotonic estimator. Note that, due to the monotonicity of the true cumulative incidence, $\sup_{x\leq t} \left[ F_{1}\left( x \right) \right]=F_{1}\left( t \right)$ and, using the boundedness of $\hat{F}_{1}\left( x \right)$ and $F_{1}\left( t \right)$ along with the supremum inequality $\sup\left( f \right)-\sup\left( g \right)\leq\sup\left( f-g \right)$, we have that

$$C_{n}\left( t \right)\leq\hat{F}_{1}\left( t \right)-F_{1}\left( t \right)\leq\sup_{x\leq t} \left[ \hat{F}_{1}\left( x \right) \right]-F_{1}\left( t \right)\leq\sup_{x\leq t} \left[ \hat{F}_{1}\left( x \right)-F_{1}\left( x \right) \right]\Rightarrow$$

$$0\leq\sup_{x\leq t} \left[ \hat{F}_{1}\left( x \right) \right]-F_{1}\left( t \right)+\left[ -C_{n}\left( t \right) \right]\leq\sup_{x\leq t} \left[ \hat{F}_{1}\left( x \right)-F_{1}\left( x \right) \right]+\left[ -C_{n}\left( t \right) \right]\Rightarrow$$

$$\left| \sup_{x\leq t} \left[ \hat{F}_{1}\left( x \right) \right]-F_{1}\left( t \right)+\left[ -C_{n}\left( t \right) \right] \right|\leq\left| \sup_{x\leq t} \left[ \hat{F}_{1}\left( x \right)-F_{1}\left( x \right) \right]+\left[ -C_{n}\left( t \right) \right] \right|,$$

where $C_{n}\left( t \right)=\min\left\{ w_{1}\left[ \hat{F}_{1}^{obs}\left( t \right)-F_{1}^{obs}\left( t \right) \right]-w_{2}\left[ {\hat{F}_{2}^{obs}\left( t \right)-F}_{2}^{obs}\left( t \right) \right],0 \right\}$ (which is bounded since all of its arguments are bounded), and the above inequality holds for all $t\in[0,\max\left( t \right)]$. Consequently,

$$\left\| \sup_{x\leq t} \left[ \hat{F}_{1}\left( x \right) \right]-F_{1}\left( t \right)+\left[ -C_{n}\left( t \right) \right] \right\|_{\infty}\leq\left\| \sup_{x\leq t} \left[ \hat{F}_{1}\left( x \right)-F_{1}\left( x \right) \right]+\left[ -C_{n}\left( t \right) \right] \right\|_{\infty}$$

$$\leq\left\| \sup_{x\leq t} \left[ \hat{F}_{1}\left( x \right)-F_{1}\left( x \right) \right] \right\|_{\infty}+\left\| -C_{n}\left( t \right) \right\|_{\infty}=\left\| \hat{F}_{1}\left( t \right)-F_{1}\left( t \right) \right\|_{\infty}+\left\| -C_{n}\left( t \right) \right\|_{\infty}.$$

Due to the uniform consistency of $\hat{F}_{1}^{obs}\left( t \right)$ and $\hat{F}_{2}^{obs}\left( t \right)$, Slutsky’s theorem and the uniform consistency of the corrected non-monotonic estimator $\hat{F}_{1}\left( t \right)$, $\left\| -C_{n}\left( t \right) \right\|_{\infty}=\left\| C_{n}\left( t \right) \right\|_{\infty}\underset{\to}{p}0$ and $\left\| \hat{F}_{1}\left( t \right)-F_{1}\left( t \right) \right\|_{\infty}\underset{\to}{p}0$. Thus, again by the Slutsky’s theorem and the above inequality, it holds that $\left\| \sup_{x\leq t} \left[ \hat{F}_{1}\left( x \right) \right]-F_{1}\left( t \right)+\left[ -C_{n}\left( t \right) \right] \right\|_{\infty}\underset{\to}{p}0$. Finally,

$$\left\| \sup_{x\leq t} \left[ \hat{F}_{1}\left( x \right) \right]-F_{1}\left( t \right)+\left[ -C_{n}\left( t \right) \right] \right\|_{\infty}=\left\| \sup_{x\leq t} \left[ \hat{F}_{1}\left( x \right) \right]-F_{1}\left( t \right)-C_{n}\left( t \right) \right\|_{\infty}\geq\left\| \sup_{x\leq t} \left[ \hat{F}_{1}\left( x \right) \right]-F_{1}\left( t \right) \right\|_{\infty}-\left\| C_{n}\left( t \right) \right\|_{\infty}$$

$$⇕$$

$$\left\| \sup_{x\leq t} \left[ \hat{F}_{1}\left( x \right) \right]-F_{1}\left( t \right) \right\|_{\infty}\leq\left\| \sup_{x\leq t} \left[ \hat{F}_{1}\left( x \right) \right]-F_{1}\left( t \right)-C_{n}\left( t \right) \right\|_{\infty}+\left\| C_{n}\left( t \right) \right\|_{\infty}.$$

By the uniform consistency of the supremum norms in the right side of the above inequality and by Slutsky’s theorem it follows straightforwardly that

$$\left\| \sup_{x\leq t} \left[ \hat{F}_{1}\left( x \right) \right]-F_{1}\left( t \right) \right\|_{\infty}\equiv\left\| \hat{F}_{1}^{m}\left( t \right)-F_{1}\left( t \right) \right\|_{\infty}\underset{\to}{p}0$$

and thus the monotonic corrected estimator of the cumulative incidence function is uniformly consistent for the true cumulative incidence of the corresponding cause of failure. This completes the proof.

*Mathematically permissible ranges for misclassification probabilities*

Let us finally prove the mathematically permissible ranges for the misclassification probabilities that can be used in a sensitivity analysis.

Since the cumulative incidence must satisfy the fundamental inequality:

$0\leq F_{1}\left( t \right)<1-S\left( t \right)$*,*

where $F_{1}\left( t \right)$ is the cumulative incidence function for the event of interest and $S\left( t \right)$ is the usual survival probability, the possible ranges for the misclassifications probabilities $a$ and $b$ under any misclassification setting (i.e. unidirectional or bidirectional) are restricted. Based on the left side of the above inequality, the relation $S\left( t \right)=1-\left[ F_{1}\left( t \right)+F_{2}\left( t \right) \right]$ and some elementary algebra on the definition of the observable cumulative incidence we have:

$F_{1}\left( t \right)\geq0\Rightarrow\frac{F_{1}^{obs}\left( t \right)-b\left[ 1-S\left( t \right) \right]}{1-a-b}\geq0.$ $(A.5)$

Since the sum of the two misclassification probabilities must not exceed 1 (i.e. 100%) we have

$$F_{1}^{obs}\left( t \right)-b\left[ 1-S\left( t \right) \right]\geq0$$

which implies

$$F_{1}^{obs}\left( t \right)\geq b\left[ 1-S\left( t \right) \right],$$

so that, in turn,

$$b\leq\frac{F_{1}^{obs}\left( t \right)}{\left[ 1-S\left( t \right) \right]}.$$

Taking into account the right side of the initial inequality (A.5) above and considering the fact that $F_{1}\left( t \right)<1-S\left( t \right)$we obtain

$$\frac{F_{1}^{obs}\left( t \right)-b\left[ 1-S\left( t \right) \right]}{1-a-b}<1-S\left( t \right),$$

which, after some algebra, results in

$$a<1-\frac{F_{1}^{obs}\left( t \right)}{1-S\left( t \right)}.$$

Consequently, the possible ranges for the misclassification probabilities are:

$$0\leq a<1-\frac{F_{1}^{obs}\left( t \right)}{1-S\left( t \right)}$$

and

$$0\leq b<\frac{F_{1}^{obs}\left( t \right)}{1-S\left( t \right)}.$$

Clearly the potential ranges of the misclassification probabilities depend on $F_{1}^{obs}\left( t \right)$ and $1-S\left( t \right)$ within the study in question. This means ranges of misclassification probabilities may substantially differ among different studies. Also, when performing sensitivity analyses, the assumed values for the potential misclassification probabilities must lie within the above ranges.

**Simulation study design**

The assumed cumulative incidence of the event of interest was assumed to arise from an exponential probability distribution:

$F_{1}\left( t \right)=0.5\left[ 1-\exp\left( -h_{1}t \right) \right]$,

Where $h_{1}$ is a hazard parameter. Based on the above cumulative incidence, the cause of failure $C$ was set equal to 1 with probability 0.5, or equal to 2 otherwise. Event time was simulated conditionally on the event type from the exponential probability distribution function:

$$\text{Pr}\left( T\leq t|C=1 \right)=1-\exp\left( -h_{1}t \right)$$

for the event of interest and from

$$\text{Pr}\left( T\leq t|C=2 \right)=1-\exp\left( -h_{2}t \right)$$

for the competing event. Hazard parameter for the cause of interest was assumed to be of medium magnitude (i.e. $h_{1}$=0.5) and for the cometing risk ($h_{2}$) was considered to be low (0.25), moderate (0.5) or high (1) depending on the scenario. Misclassification probabilities $a$ and $b$ were considered to be equal to 0%, 10%, 20% or 30%. The observable and possibly misclassified event type $C^{obs}$ was set equal to $C$ with probability $1-a$ (for cases with $C=1$) and $1-b$ (for cases with $C=2$), or different to *C* (i.e. misclassification) otherwise. Right censoring was simulated from the uniform distribution $U(0,7)$. The combination of the possible parameter values resulted in a total of 45 simulation scenarios (excluding scenarios with both $a=0$ and $b=0$).

**References**

Andersen, P. K., Borgan, O., Gill, R. D. and Keiding, N. (1996). Statistical models based on counting processes. Springer-Verlag.
